# Comparative Analysis of Large Language Models in Dermatological Diagnosis: An Evaluation of Diagnostic Accuracy

**DOI:** 10.7759/cureus.92089

**Published:** 2025-09-11

**Authors:** Niharika Tekchandani, Anurup Mukherjee, Nandakumar Poonthottam, Stergios Boussios

**Affiliations:** 1 Medicine, Medway NHS Foundation Trust, Kent, GBR; 2 Digital Health/Internal Medicine, Kent and Medway Medical School/Maidstone and Tunbridge Wells NHS Trust, Kent, GBR; 3 Medical Oncology, Medway NHS Foundation Trust, Kent, GBR

**Keywords:** artificial intelligence, dermatology diagnosis, diagnostic accuracy, large language models, rare skin diseases

## Abstract

Background: The diagnostic process in dermatology often hinges on visual recognition and clinical pattern matching, making it an attractive field for the application of artificial intelligence (AI). Large language models (LLMs) like ChatGPT-4o, Claude 3.7 Sonnet, and Gemini 2.0 Flash offer new possibilities for augmenting diagnostic reasoning, particularly in rare or diagnostically challenging cases. This study evaluates and compares the diagnostic capabilities of these LLMs based solely on clinical presentations extracted from rare dermatological case reports.

Methodology: Fifteen published case reports of rare dermatological conditions were retrospectively selected. Key clinical features, excluding laboratory or histopathological findings, were input into each of the three LLMs using standardized prompts. Each model produced a most probable diagnosis and a list of differential diagnoses. The outputs were evaluated for top-match accuracy and whether the correct diagnosis was included in the differential list. Performance was analyzed descriptively, with visual aids (heatmaps, bar charts) illustrating comparative outcomes.

Results: ChatGPT-4o and Claude 3.7 Sonnet each correctly identified the top diagnosis in 10 (66.7%) out of 15 cases, compared to 8 (53.3%) out of 15 for Gemini 2.0 Flash. When differential-only matches were included, both ChatGPT-4o and Claude 3.7 achieved a total coverage of 86.7%, while Gemini 2.0 reached 60.0%. Notably, all models failed to identify certain diagnoses, including blastic plasmacytoid dendritic cell neoplasm and amelanotic melanoma, underscoring the potential risks associated with plausible but incorrect outputs.

Conclusions: This study demonstrates that ChatGPT-4o and Claude 3.7 Sonnet show promising diagnostic potential in rare dermatologic cases, outperforming Gemini 2.0 Flash in both accuracy and diagnostic breadth. While LLMs may assist in clinical reasoning, particularly in settings with limited dermatology expertise, they should be used as adjunctive tools, not substitutes, for clinician judgment. Further refinement, validation, and integration into clinical workflows are warranted.

## Introduction

The field of dermatology, characterized by its reliance on visual pattern recognition and extensive clinical experience, is increasingly being influenced by the advent of artificial intelligence (AI), specifically large language models (LLMs) [1]. The application of these models holds promise for augmenting diagnostic capabilities, streamlining clinical workflows, and ultimately improving patient care [2-5]. AI has already started transforming dermatology by improving diagnostic accuracy, treatment planning, and surgical support [4]. The integration of machine learning in healthcare offers possibilities for enhancing care across a spectrum of applications [6]. In recent years, machine learning has demonstrated semantic understanding and information extraction, sometimes detecting abstract patterns with greater accuracy than human experts. This study delves into the diagnostic performance of three prominent LLMs - ChatGPT-4o, Claude 3.7 Sonnet, and Gemini 2.0 Flash - in the context of rare dermatological conditions. We present a comparative analysis of their ability to generate accurate diagnoses and comprehensive differential diagnoses based solely on the clinical features extracted from a collection of case reports, which are the cornerstone for clinical acumen in rare dermatological diseases. The clinical integration of AI in dermatology workflow remains challenging despite its potential in medical applications [7]. The rationale behind this research stems from the growing recognition of the potential of AI to assist dermatologists in diagnostic decision-making [8]. However, the deployment of AI in daily clinical workflows is still a problem.

The primary objective of this study was to evaluate and compare the diagnostic accuracy of three LLMs: ChatGPT-4o, Claude 3.7 Sonnet, and Gemini 2.0 Flash, when applied to rare dermatological cases. For clarity, diagnostic accuracy was defined as both correct top-choice predictions and inclusion of the correct diagnosis within differential diagnosis lists. Secondary objectives included assessing their ability to generate clinically relevant differential diagnoses and analyzing case-level failures to identify contexts in which these tools may be less reliable. We focused on rare dermatological conditions because they are diagnostically challenging, often underrepresented in training corpora, and carry significant clinical implications when misdiagnosed. We hypothesized that while all three models would demonstrate some degree of diagnostic accuracy, important differences would emerge in their ability to handle rare and diagnostically complex conditions. By addressing this gap, our study provides early insights into the potential and limitations of LLMs for dermatology and highlights areas for future refinement. By addressing this gap, our study contributes to the growing body of work comparing LLMs in clinical decision-making [9] and provides early insights into their potential and limitations for dermatology. Furthermore, in line with prior work exploring AI-based tools in dermatology [10], we emphasize the importance of developing a fundamental understanding of AI within the medical community to ensure safe and effective integration into clinical workflows.

## Materials and methods

Data acquisition and preparation

A retrospective collection of 15 case reports, each representing a distinct and relatively rare dermatological condition, served as the foundation for this study [11-25]. We selected these 15 cases because they represent rare dermatological conditions documented in recent open-access case reports that provided sufficient clinical detail for standardized extraction. Many other published reports were excluded because they lacked adequate clinical descriptions to be used in our prompt template. Clinical features were transcribed verbatim from the published case reports, restricted to sections describing patient demographics (age, sex), lesion morphology (size, shape, color, distribution), and associated symptoms. Clinical features were independently extracted by two clinically trained reviewers. Discrepancies were first resolved through discussion and consensus; if consensus could not be reached, a third reviewer adjudicated to ensure accuracy and minimize bias in the data abstraction process. The small sample size reflects the relative scarcity of such published cases, but it allowed for focused evaluation of LLM performance across diverse, diagnostically challenging conditions.

Ethical considerations were prioritized by ensuring that all patient identifiers were removed from the case reports, safeguarding patient confidentiality, and adhering to established ethical guidelines for medical research. To emulate the diagnostic process initiated during a patient encounter, data extraction was performed to isolate key clinical features detailed within each case report.

The extracted features encompassed a spectrum of pertinent information, including the patient’s age, sex, detailed descriptions of lesion morphology (e.g., size, shape, color, distribution), and associated symptoms.

A standardized prompt structure was used across all models. A representative example of the prompt, together with the corresponding model output, is provided to enhance reproducibility and transparency (Appendix). All models were queried between May and June 2025 using their official web-based interfaces, ensuring consistency across outputs.

LLM prompting and output collection

The prepared clinical features for each case were then systematically input into the three LLMs under investigation: ChatGPT-4o, Claude 3.7 Sonnet, and Gemini 2.0 Flash. To ensure consistency and minimize bias, a standardized prompt structure was employed across all models. The prompts were carefully designed to elicit two distinct outputs from each model: the most probable diagnosis based on the provided clinical features, and a list of differential diagnoses.

The models’ outputs, comprising the most probable diagnosis and the list of up to five differential diagnoses for each case, were recorded and organized into a structured format suitable for subsequent analysis. This process ensured that all relevant information was captured accurately and consistently, enabling a robust comparison of the models’ diagnostic capabilities.

Evaluation metrics and statistical analysis

The accuracy of the models’ most probable diagnoses was assessed by comparing them to the confirmed diagnoses documented in the original case reports. In instances where the most probable diagnosis provided by a model did not align with the actual diagnosis, the list of differential diagnoses generated by that model was examined to determine if the correct diagnosis was included among the proposed differentials. The quality of the differential diagnosis lists was evaluated using the approach described by McDuff et al. [26], which defines diagnostic coverage as the inclusion of the correct diagnosis either as the top choice or within the list of differentials.

## Results

Table 1 presents the actual diagnoses of the 15 dermatological cases and the most probable diagnoses generated by the three LLMs under investigation.

**Table 1:** Comparison of actual diagnoses and model-predicted top diagnoses across 15 rare dermatological cases.

Case no.	Actual diagnosis	ChatGPT-4o	Claude 3.7 Sonnet	Gemini 2.0 Flash
1	Cutaneous TB	Cutaneous tuberculosis	Tuberculosis cutis colliquativa (scrofuloderma)	Atypical mycobacterial infection (cutaneous)
2	Pachydermodactyly	Pachydermodactyly	Pachydermodactyly	Digital knuckle pads
3	Blastic Plasmacytoid Dendritic Cell Neoplasm	Cutaneous metastasis of Merkel cell carcinoma (MCC)	Coumarin (warfarin) necrosis with an associated basal cell carcinoma on the right thigh	Amyloidosis (cutaneous involvement) with potential for systemic involvement
4	Granulomatosis With Polyangiitis	Granulomatosis with polyangiitis (GPA)	Granulomatosis with Polyangiitis (GPA)	Granulomatosis with Polyangiitis (GPA)
5	Atrophoderma of Pasini and Pierini	Atrophoderma of Pasini and Pierini (APP)	Lichen Sclerosus et Atrophicus	Atrophoderma of Pasini and Pierini
6	Cutaneous Endometriosis Arising in a Cesarean Scar	Scar endometriosis	Cutaneous Endometriosis (also known as Scar Endometriosis)	Cutaneous endometriosis
7	Hidrotic ectodermal dysplasias (Clouston syndrome)	Hereditary Hypotrichosis Simplex with Nail Dystrophy	For this 45-year-old female is Hidrotic Ectodermal Dysplasia (Clouston syndrome)	Hidrotic ectodermal dysplasia (Clouston syndrome)
8	Atypical Mal de Meleda in a Hispanic Patient	Mal de Meleda	Mal de Meleda (MDM)	Vohwinkel syndrome (Mitten hand and stocking feet hyperkeratosis with pseudoainhum and sensorineural deafness
9	LEOPARD Syndrome with a Sporadic PTPN11 Mutation in a Saudi Patient	LEOPARD syndrome (now often grouped under Noonan syndrome with multiple lentigines	LEOPARD syndrome (Noonan syndrome with multiple lentigines)	LEOPARD syndrome
10	Confluent and reticulated papillomatosis (CARP)	Pityriasis versicolor	Tinea versicolor (pityriasis versicolor)	Tinea versicolor (Pityriasis versicolor)
11	Hypopigmented Mycosis Fungoides in an 11-Year-Old Palestinian Boy	Hypopigmented Mycosis Fungoides (HMF)	Hypopigmented mycosis fungoides	Mycosis fungoides
12	Granulosis Rubra Nasi	For both patients, it is an Eccrine Angiomatous Hamartoma (EAH)	For both cases is granulosis rubra nasi	Lupus miliaris disseminatus faciei (LMDF)
13	Acquired Lymphangiectasia of the Scrotum	Scrotal Lymphangioma Circumscriptum	Scrotal lymphangioma circumscriptum	Scrotal lymphangioma circumscriptum
14	A Rare Cutaneous Manifestation: Leukocytoclastic Vasculitis after Pfizer-BioNTech COVID-19 Vaccination	Leukocytoclastic vasculitis (LCV)	COVID-19 vaccine-induced immune thrombocytopenic purpura (ITP)	Vaccine-induced immune thrombocytopenia (VITT) or a cutaneous small vessel vasculitis secondary to the Pfizer-BioNTech COVID-19 booster vaccine
15	Amelanotic Melanoma	Intraepidermal (cutaneous) squamous cell carcinoma in situ	Porokeratosis of Mibelli	Atypical squamous cell carcinoma in situ (Bowen's disease) or a superficial basal cell carcinoma

A crucial performance indicator was the *Top Matches* metric, which quantified the number of cases in which the model correctly identified the most probable diagnosis. In addition to evaluating the models’ ability to pinpoint the correct diagnosis, the research also delved into their capacity to generate comprehensive and accurate differential diagnosis lists.

The performance summary table below summarizes an evaluation of the diagnostic capabilities of three LLMs in the context of 15 dermatological cases (Table 2). The analysis examined whether the models’ most probable diagnoses aligned with the actual case diagnoses, and whether the correct diagnosis was included in the models’ differential diagnosis lists.

**Table 2:** Diagnostic performance summary of LLMs across 15 dermatological cases. LLM, large language model

Model	Top matches, *n* (%)	Differential matches (only), *n* (%)	Total coverage (Top or Diff), *n* (%)
ChatGPT-4o	10 (66.7%)	3 (20.0%)	13 (86.7%)
Claude 3.7 Sonnet	10 (66.7%)	3 (20.0%)	13 (86. 7%)
Gemini 2.0 Flash	8 (53.3%)	1 (6.7%)	9 (60.0%)

**Figure 1:**
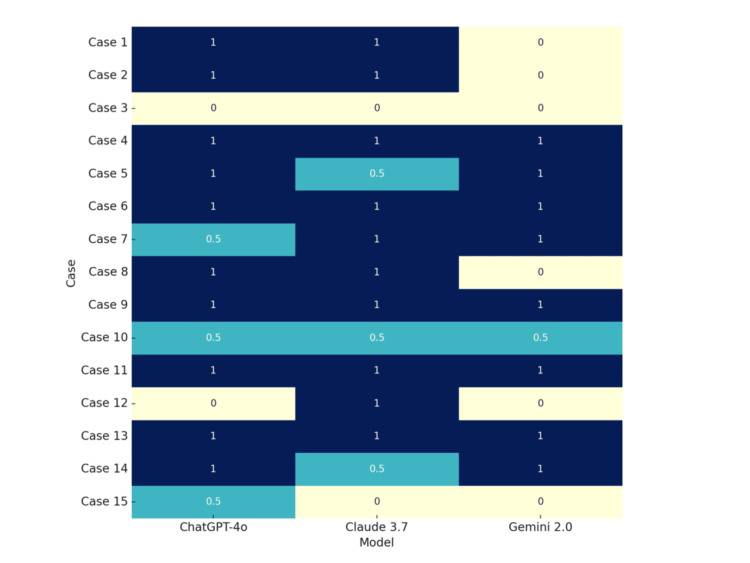
Heatmap of diagnostic accuracy across 15 cases (1 = correct top match, 0.5 = differential match, 0 = miss).

The heatmap below provides a visual representation of the models’ performance across individual cases (Figure 1). A value of 1 signifies that the model correctly identified the diagnosis as the top choice, while a value of 0.5 indicates that the correct diagnosis was included in the list of differential diagnoses. Conversely, a value of 0 denotes that the model failed to accurately identify the correct diagnosis.

In terms of diagnostic accuracy, ChatGPT-4o and Claude 3.7 Sonnet each correctly identified the most probable diagnosis in 10 (66.7%) of 15 cases, while Gemini 2.0 Flash achieved a lower top-match rate of 8 out of 15 (53.3%). When including cases where the correct diagnosis was not the top prediction but appeared in the list of differentials, both ChatGPT-4o and Claude 3.7 Sonnet demonstrated a total coverage rate of 86.7%, reflecting a broader diagnostic awareness. In contrast, Gemini 2.0 Flash showed limited differential utility, reaching a total coverage of 60.0%, with only 1 case out of 15 (6.7%) containing the correct diagnosis in the differential list without it being the top choice. These results highlight that while Gemini demonstrated some diagnostic capability, ChatGPT-4o and Claude 3.7 provided substantially more reliable outputs, both in primary and supporting diagnoses.

**Figure 2:**
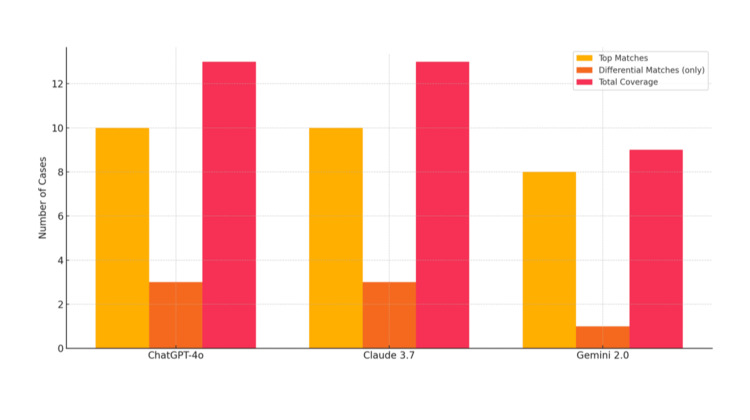
Comparison of top match, differential-only, and total diagnostic coverage per LLM. LLM, large language model

The bar chart below presented compares the overall performance of the three language models in terms of the total number of top matches, differential-only matches, and overall coverage (Figure 2). The results indicate that ChatGPT-4o and Claude 3.7 Sonnet demonstrate comparable total coverage, although their individual strengths may differ.

Noteworthy findings include five cases in which all three models unanimously succeeded in identifying the correct diagnosis. In contrast, Case 3, involving Blastic Plasmacytoid Dendritic Cell Neoplasm (BPDCN), was a notable failure, with all models inaccurately predicting the diagnosis. Neither ChatGPT-4o nor Gemini 2.0 Flash demonstrated any exclusive instances of outperforming both of the other models. However, Claude 3.7 Sonnet showed one exclusive instance of superior performance in Case 12, correctly identifying Granulosis Rubra Nasi when the other models did not.

The small sample size and paired nature of the data limited the reliability of a chi-square test. Given these constraints, descriptive statistics and visual comparisons were prioritized over inferential tests. Future studies with larger case sets may permit more robust statistical comparisons using paired testing methods such as McNemar’s test.

The results revealed that ChatGPT-4o and Claude 3.7 Sonnet exhibited similar levels of overall accuracy, outperforming Gemini 2.0 Flash in terms of both top matches and differential diagnoses. The analysis indicated that while all three models demonstrated proficiency in certain cases, there were also instances where they struggled to accurately identify the correct diagnosis.

## Discussion

The integration of LLMs into dermatological diagnostics presents an exciting advancement in AI-assisted healthcare. In this study, we evaluated the performance of three advanced LLMs - ChatGPT-4o, Claude 3.7 Sonnet, and Gemini 2.0 Flash across 15 rare dermatological cases, assessing their ability to generate accurate primary diagnoses and plausible differential lists based solely on clinical findings.

Overall, ChatGPT-4o and Claude 3.7 demonstrated strong diagnostic capabilities, correctly identifying the top diagnosis in 10 of 15 cases (66.7%) and achieving a total diagnostic coverage of 13 of 15 (86.7%) cases when differentials were included. Gemini 2.0 Flash, while still competent, lagged slightly behind with a top match accuracy of 8 (53.3%) of 15 cases and total coverage of 9 (60.0%) of 15 cases. These results highlight the potential utility of certain LLMs in clinical decision support, particularly in settings where immediate dermatological expertise may not be available.

However, aggregate accuracy does not tell the full story. Specific case-level failures illuminate important limitations. In Case 3, for example, BPDCN - all three models failed to identify the diagnosis, either as a top choice or within their differential lists. This suggests that LLMs, while broadly informed, may struggle with rare hematologic-dermatologic interface disorders that require domain-specific training. In another critical failure (Case 15), the correct diagnosis of amelanotic melanoma was missed by all models, with plausible but incorrect suggestions such as squamous cell carcinoma or benign inflammatory conditions offered instead. Both conditions are rare and likely underrepresented in training corpora. In addition, BPDCN requires recognition of complex hematologic-dermatologic overlap, while amelanotic melanoma depends heavily on visual cues such as dermoscopic features, which are difficult to capture in text-only prompts. These errors underscore the risk that confident, medically reasonable but inaccurate outputs may create a false sense of diagnostic certainty for clinicians [27].

Descriptive statistics and visual comparisons (e.g., heatmaps, bar charts) provided valuable insight into performance differences between models. While ChatGPT-4o and Claude 3.7 Sonnet showed superior diagnostic alignment, model performance varied across cases, emphasizing the importance of case complexity and presentation in influencing outcomes. The inclusion of differential diagnoses proved to be a useful metric of diagnostic awareness, particularly in scenarios where the top diagnosis was incorrect.

Importantly, the clinical relevance of these findings extends into both general and aesthetic dermatology. In cosmetic practice, where patients frequently present with pigmentary or textural concerns, distinguishing benign aesthetic issues from serious pathologies is critical. LLMs may support practitioners by flagging uncommon but clinically significant conditions early in the diagnostic process. Their ability to synthesize differential diagnoses also supports patient triage, referral decisions, and pre-procedural screening, enhancing safety and clinical efficiency.

Despite promising results, careful consideration must be given to the risks of over-reliance on LLMs. These tools should be viewed as augmented intelligence, not replacements for clinical judgment. Misleading confidence, knowledge gaps, and hallucinated details remain nontrivial risks. To safely implement LLMs in dermatology, future efforts must include robust validation, transparent uncertainty estimation, and training on diverse, representative datasets [27][1]. Additionally, prompt engineering and model fine-tuning tailored to dermatological use cases could significantly improve reliability [27].

The performance of large language models in this study highlights their potential as diagnostic aids, while also underscoring the importance of careful evaluation and validation. The performance of these models is influenced by the quality and format of the input data. These models may be particularly helpful in rare and difficult-to-diagnose cases, where clinical experience and expert knowledge are critical.

In summary, while ChatGPT-4o and Claude 3.7 Sonnet show strong potential as clinical diagnostic assistants, critical evaluation of their outputs and caution in their application remains essential. With proper safeguards, LLMs may evolve into powerful tools that support, rather than supplant, the clinical expertise at the heart of dermatologic care.

The study underscores the importance of validating LLMs on diverse and representative datasets, as well as establishing clear guidelines for their responsible and ethical implementation in healthcare settings [28-29].

Limitations

This study has several limitations. The sample size was small, comprising only 15 cases, which restricts the generalizability of the findings. Because the cases were drawn from published reports of rare dermatological conditions, selection bias is possible, as such reports may overrepresent diagnostically complex or unusual presentations. The rarity of the included conditions also limits their representativeness for routine dermatology practice. In addition, only clinical features were provided to the models, without histopathological or dermoscopic data. This introduces a fundamental visual-textual disconnect, given that dermatology is primarily a visual discipline, and limits the clinical applicability of our findings, particularly for conditions such as melanoma. Diagnostic outputs were further influenced by the specific prompt structure, as prompt engineering can significantly affect performance. Moreover, the LLMs assessed were trained primarily on general-domain data rather than dermatology-specific corpora, which may constrain their accuracy in highly specialized cases. As LLMs are non-static systems that evolve with ongoing updates, strict reproducibility of results cannot be guaranteed even when identical prompts are used. Finally, because this was a retrospective analysis of published cases, it cannot account for real-time clinical variability. Prospective studies incorporating larger, more representative datasets and multimodal inputs will be essential to establish the clinical utility of these tools.

## Conclusions

This study directly compared the diagnostic accuracy of three large language models, namely, ChatGPT-4o, Claude 3.7 Sonnet, and Gemini 2.0 Flash in rare dermatological cases. ChatGPT-4o and Claude 3.7 Sonnet outperformed Gemini 2.0 Flash in both top-match accuracy and total diagnostic coverage, although performance varied across cases depending on complexity and clinical presentation. Because dermatology is fundamentally a visual specialty, the reliance on text-based case descriptions represents a key limitation that constrains clinical applicability. Model predictions were compared against gold-standard diagnoses established in the published case reports, rather than through direct benchmarking against clinician performance, and the findings should therefore be interpreted as preliminary and exploratory. While these results suggest that certain LLMs hold promise as diagnostic aids, careful evaluation is essential for responsible implementation. Future research including multimodal approaches that incorporate visual data and prospective benchmarking against dermatologist performance will be critical to establish the true clinical utility of these tools in dermatology and other medical specialties.

## Appendices

Appendix

Representative Prompt (ChatGPT-4o):

“You are an expert dermatologist. Based on the following clinical features, please provide only the names of the most probable diagnosis and a list of up to five differential diagnoses.

Clinical features: A 24-year-old Hispanic woman presented with a painful lesion on her right buttock that began during her pregnancy four years prior. The lesion appeared as a large, reddish-brown, scaly plaque with well-defined borders and central atrophic changes covering the entire surface of the right buttock. The lesion was tender and warm with notable expression to light touch of purulent material through multiple fissures along the periphery. The patient reported no other symptoms, such as fever, chills, cough, or fatigue. On physical examination, vital signs were within normal limits, the skin demonstrated no other significant changes, and the patient had no notable lymphadenopathy. On history, the patient reported the skin lesion had progressively increased over the past four years. She had been previously diagnosed with psoriasis and was treated with multiple topical therapies, including salicylic acid and potent topical corticosteroids without any relief. Further, the patient reported having a similar lesion (which was on her neck) as a child that was surgically removed in Mexico. [11]”

ChatGPT-4o Response:

Most Probable Diagnosis: Cutaneous tuberculosis

Differential Diagnoses:

1. Deep fungal infection (e.g., blastomycosis, sporotrichosis)

2. Cutaneous sarcoidosis

3. Chronic cutaneous leishmaniasis

4. Hypertrophic lichen planus

5. Chronic plaque psoriasis with secondary infection

Note: The model also suggested that histopathological or microbiological workup would be useful for confirmation.
